# Whole-body simultaneous time-of-flight PET-MRI: early experience with clinical studies

**DOI:** 10.1186/2197-7364-2-S1-A64

**Published:** 2015-05-18

**Authors:** Ryogo Minamimoto, Andrei Iagaru, Mehran Jamali, Amir Barkodhodari, Dawn Holley, Shreyas Vasanawala, Greg Zaharchuk

**Affiliations:** Stanford University, Department of Radiology, Division of Nuclear Medicine and Molecular Imaging, USA

Recently, a whole-body, simultaneous positron emission tomography - magnetic resonance imaging (PET-MRI) system combing MRI with time-of-flight (TOF) PET has been developed. We present our initial experience with human clinical studies using 18Ffluorodeoxyglucase (FDG) with this scanner, in comparison to PET-CT. All patients underwent a single-injection of 18F-FDG, with a dual-imaging protocol consisting of PET-CT followed by PET-MRI scan. PET-MR attenuation correction used a two point Dixon fat-water separated method for the body, combined with registration to an atlas for the head. Two radiologists evaluated MRI image quality using the following scale (0 non-diagnostic; 1 poor; 2 good; 3 excellent). PET-MRI and PET-CT images were reviewed for FDG uptake thought to be consistent with malignancy by two readers independently, and categorized into 5 groups (1 both PETMRI and PET-CT positive, 2 PET-MRI positive, PET-CT positive in retrospect; 3 PET-CT positive, PET-MRI positive in retrospect; 4: PET-MRI positive, PET-CT negative; 5: PET-MRI negative, PET-CT positive) by consensus.

## Results

Twenty-six oncologic patients (average age: 63±14 yrs) with were enrolled in the study. PET-CT and PETMRI scan started 71±14 and 144±22 minutes after injection of 10Å}1 mCi FDG, respectively. The average length of the PET-CT and PET-MRI scan was 20±7, and 55±15 minutes, respectively. All MRI images were rated to be diagnostic; 64% were rated excellent, 32% were rated good, and 4% were rated poor. 64% (34/53) of FDG intense lesions were observed in the same location for both PET-CT and PET-MRI. TOF PET-MRI provided comparable image quality and diagnostic ability with PET-CT, despite imaging at a later time point.

